# α2AP mediated myofibroblast formation and the development of renal fibrosis in unilateral ureteral obstruction

**DOI:** 10.1038/srep05967

**Published:** 2014-08-06

**Authors:** Yosuke Kanno, Eri Kawashita, Akiko Kokado, Hiromi Kuretake, Kanako Ikeda, Kiyotaka Okada, Mariko Seishima, Shigeru Ueshima, Osamu Matsuo, Hiroyuki Matsuno

**Affiliations:** 1 Department of Clinical Pathological Biochemistry, Faculty of Pharmaceutical Science, Doshisha Women's Collage of Liberal Arts, 97-1 Kodo Kyo-tanabe, Kyoto, 610-0395, Japan; 2 Department of Physiology II. Kinki University School of Medicine, Osaka-sayama, Japan; 3 Department of Dermatology, Gifu University Graduate School of Medicine, Yanagido Gifu, Japan; 4 Department of Food Science and Nutrition, Kinki University School of Agriculture, Nara, Japan

## Abstract

Renal fibrosis is the final common pathway of a wide variety of chronic kidney diseases. Myofibroblast formation via the differentiation of from tissue-resident fibroblasts and bone marrow-derived mesenchymal stem cells (MSCs), and epithelial-to-mesenchymal transition (EMT) is known to play a pivotal role in the development of renal fibrosis. However, the detailed mechanisms underlying this disorder remain unclear. We herein investigated the role of alpha 2-antiplasmin (α2AP) in myofibroblast formation and the development of renal fibrosis. We observed the development of renal fibrosis using unilateral ureteral obstruction (UUO). α2AP had accumulated in the UUO-induced obstructed kidneys and α2AP deficiency attenuated UUO-induced renal fibrosis in mice. The degree of myofibroblast formation in the obstructed kidneys of α2AP^−/−^ mice was less than that in α2AP^+/+^ mice. In vitro, α2AP induced myofibroblast formation in renal tubular epithelial cells (RTECs), renal fibrosblasts, and bone marrow-derived mesenchymal stem cells (MSCs). α2AP also induced the production of TGF-β, which is known to be a key regulator of myofibroblast formation and fibrosis. α2AP-induced the TGF-β production was significantly reduced by SP600125, c-Jun N-terminal kinase (JNK) specific inhibitor. Our findings suggest that α2AP induces myofibroblast formation in the obstructed kidneys, and mediates the development of renal fibrosis.

Renal fibrosis is the final common pathway of chronic kidney diseases including diabetic nephropathy and glomerulonephritis, and is characterized by the excessive production, deposition, and contraction of the extracellular matrix (ECM). Renal fibrosis represents one of the largest groups of disorders for which there is no effective therapy. The lack of appropriate antifibrotic therapy arises primarily from the fact that the etiology of renal fibrosis is unknown. The development of renal fibrosis is generally considered to result from maladaptive repair processes induced by the release of a variety of profibrotic factors such as transforming growth factor-beta (TGF-β), in which infiltrating inflammatory cells including macrophages, stimulate the formation of myofibroblasts via the differentiation from tissue-resident fibroblasts and bone marrow-derived mesenchymal stem cells (MSCs), and epithelial-to-mesenchymal transition (EMT). The accumulated myofibroblasts subsequently synthesize and deposit components of the extracellular matrix (ECM)^1^,^2^,^3^,^4^.

Alpha2-antiplasmin (α2AP) is a serine protease inhibitor (serpin) with a molecular weight of 65 to 70 kDa^5^ that rapidly inactivates plasmin, thus resulting in the formation of a stable inactive complex, plasmin-α2AP^6^. Many studies have reported that the levels of the plasmin-α2AP complex in the plasma are elevated in patients with fibrotic diseases, including diabetic nephropathy, systemic sclerosis, liver cirrhosis and rheumatoid arthritis^7^,^8^,^9^,^10^. Recently, we found that α2AP is associated with wound healing^11^ and the development of dermal fibrosis^12^,^13^,^14^. We also demonstrated that α2AP induces the production of TGF-β, which is known to be a key regulator of the formation of myofibroblasts and the development of fibrosis. We herein report the role of α2AP in the formation of myofibroblasts and the development of renal fibrosis.

## Methods

The animal experiments in this study were approved by the Animal Research Committee of Doshisha Women's Collage of Liberal Arts (Approval ID: Y13-017).

### Animals

Deficient mice were generated by homologous recombination using embryonic stem cells, as described previously^15^,^16^. All experiments were performed in accordance with institutional guidelines.

### Reagents

α2AP was purchased from Calbiochem (CA, USA). Other chemical substances were obtained from Sigma (MO, USA).

### Unilateral ureteral obstruction (UUO)

UUO was performed as described by Miyajima et al^17^. Male mice 8 to 12 weeks of ages were used for the experiments. The left ureter of each mouse was ligated under general anesthesia. The degree of renal injury was studied at 2 or 7 days after UUO (n = 4 mice per group).

### Collagen content in kidney (The sircol biochemical assay)

The collagen content was measured as previously described^18^. The collagen content was assessed using Sirius red staining. In these assays, sections are stained with Sirius red as described by Junqueira et al^19^. After deparaffinization, the sections are treated in 0.2% phosphomolybdic acid for 5 minutes. Next, the section stained in 0.1% Sirius red for 90 minutes and 0.01 N HCl for 2 minutes. The stained images obtained from separate fields on the specimens (n = 4) were analyzed by using ImageJ. Sirius red positive area was expressed as a percent of the observed with sham mice.

### Primary murine renal tubular epithelial cells

Primary murine renal tubular epithelial cells were obtained as described by Sato et al^20^. The minced kidneys were washed with three changes of cold PBS containing 1 mM EDTA and digested in 0.25% trypsin solution in a shaking incubator at 37°C for 2 hours. Trypsin was neutralized with Dulbecco's modified Eagle medium (DMEM) containing 10% fetal calf serum (FCS). The suspension was triturated using pipetting and passed through a 70 μm cell strainer. The cells were seeded onto 60-mm diameter dishes and maintained in DMEM containing 10% FCS at 37°C in a humidified atmosphere of 5% CO_2_/95% air. The experiments were carried out in serum-free DMEM.

### Primary murine renal fibroblasts

Primary murine renal fibroblasts were obtained as described by Muller et al^21^. The renal cortex was dissected from the kidney, minced and suspended in DMEM containing 10% FCS. The cells were cultured in DMEM containing 10% fetal calf serum (FCS) at 37°C in a humidified atmosphere of 5% CO_2_/95% air, and after two to three passages, only fibroblasts survived under the culture conditions. The cells were seeded onto 60-mm diameter dishes, and maintained in DMEM containing 10% FCS at 37°C in a humidified atmosphere of 5% CO_2_/95% air. The experiments were carried out in serum-free DMEM.

### Mesenchymal stem cell isolation, cells culture

Bone marrow cells were obtained as described by Kanno et al^22^. The cells were seeded, and then were maintained in DMEM containing 10% fetal calf serum (FCS) at 37°C in a humidified atmosphere of 5% CO_2_/95% air. Nonadherent cells were removed by 2–3 washes with PBS and adherent cells further cultured in DMEM containing 10% fetal calf serum (FCS).

### Western blot analysis

We studied a Western blot analysis as previously described^23^. We detected α2AP, α-SMA, E-cadherin and vimentin, TGF-β by incubation with anti-α2AP antibody, anti-α-SMA antibody, anti-E-cadherin antibody, anti-vimentin antibody and anti-TGF-β antibody followed incubation with horseradish peroxidase-conjugated antibody to rabbit IgG.

### Statistical analysis

All data are expressed as mean ± SEM. The significance of the effect of each treatment (*P* < 0.05) was determined by analysis of variance (ANOVA) followed by the Least significant difference test.

## Results

### The accumulation of α2AP was induced in the obstructed kidney

**Figure 1:**
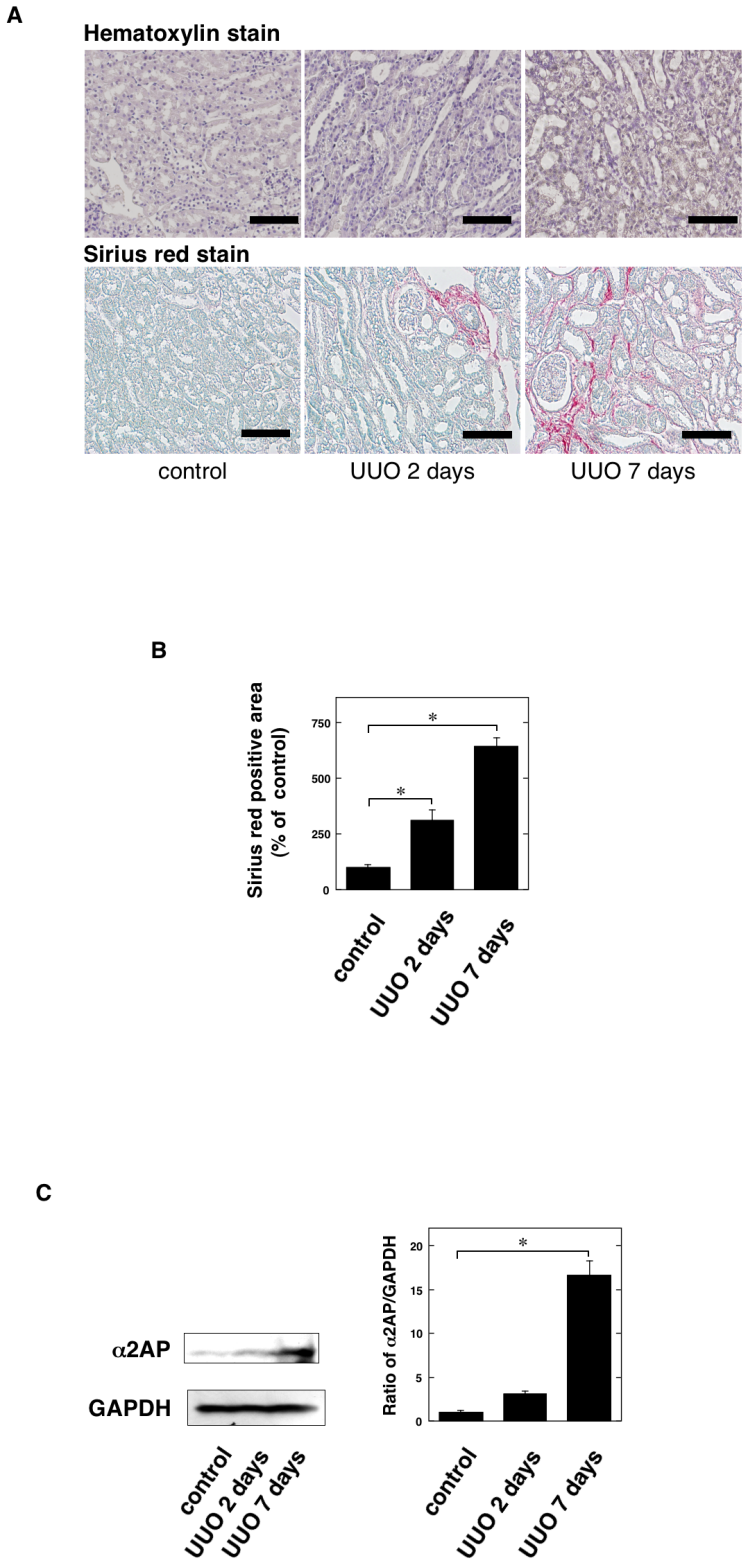
The accumulation of α2AP was induced in the obstructed kidneys. (A) The kidneys at 2 and 7 days after UUO in the wild-type mice (hematoxylin stain and Sirius red stain). (B) The collagen content measured by Sirius red stain in the kidney of control and 2 and 7 days after UUO in the wild-type mice (n = 4). Sirius red positive area was expressed as a percent of the observed with control in the wild-type mice. (C) The expression of α2AP in the kidney of control and 2 and 7 days after UUO in mice was measured by a Western blot analysis. The blots were cropped, and the full-length blots are presented in the supplementary information. The histogram on the right panels shows quantitative representations of α2AP expression obtained from densitometry analysis (n = 3). The data represent the mean ± SEM. *; *P* < 0.01. Scale bar = 100 μm.

We assessed the levels of UUO-induced renal fibrosis using Sirius red staining. The degree of Sirius red-positive areas in the obstructed kidneys at 7 days was significantly increased in comparison to that observed in the controls (Fig. 1A, B). To clarify the role of α2AP in renal fibrosis, we examined the expression of α2AP in the kidneys following UUO surgery by a Western blot analysis. We found that α2AP had accumulated in the obstructed kidneys (Fig. 1C).

### α2AP deficiency attenuated UUO-induced renal fibrosis

**Figure 2:**
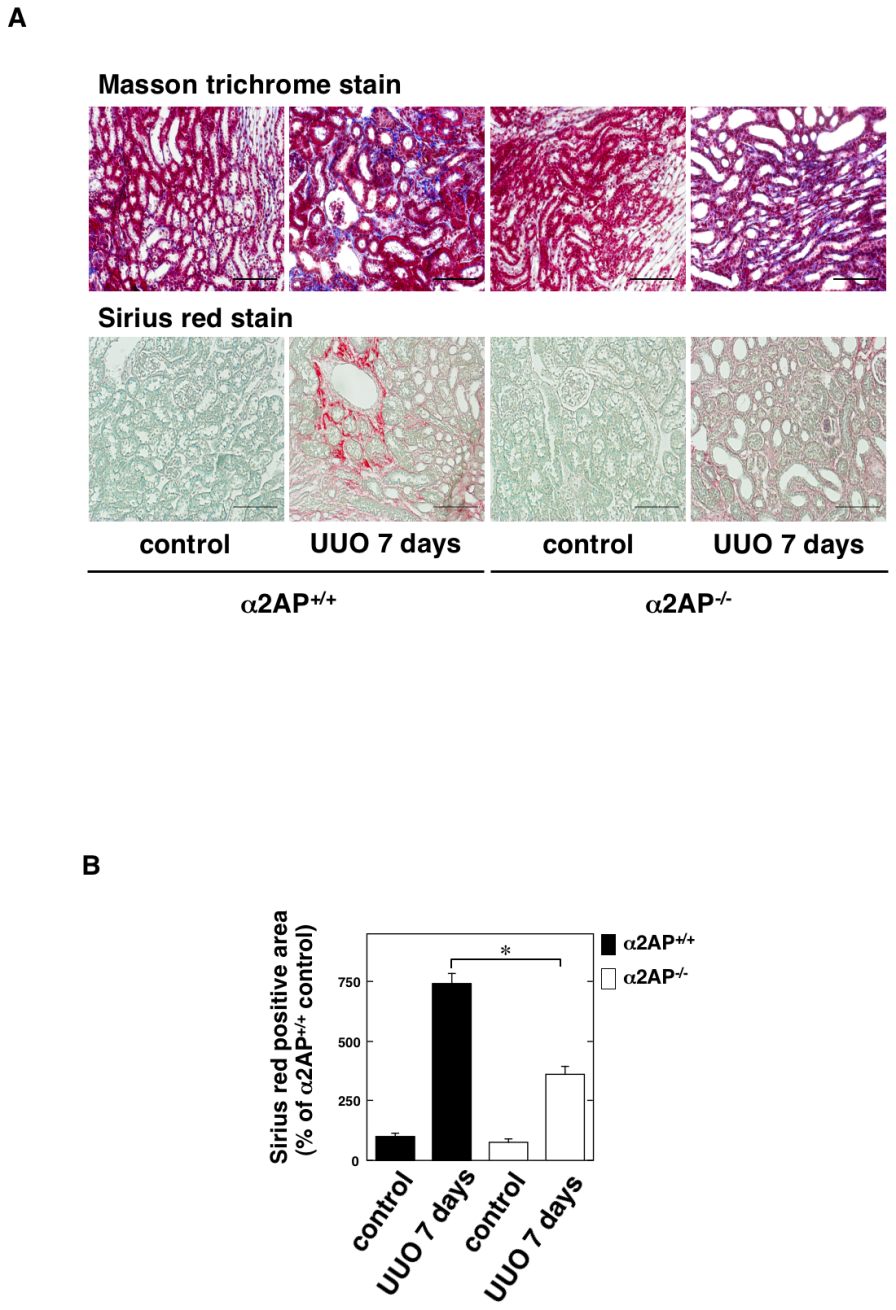
α2AP deficiency attenuated UUO-induced renal fibrosis. (A) The kidneys at 7 days after UUO in the α2AP^+/+^ and α2AP^−/−^ mice (Masson trichrome stain and Sirius red stain). (B) The collagen content measured by Sirius red stain in the kidneys of control and 7 days after UUO in the α2AP^+/+^ and α2AP^−/−^ mice (n = 4). Sirius red positive area was expressed as a percent of the observed with control in the α2AP^+/+^ mice. The data represent the mean ± SEM. *; *P* < 0.01. Scale bar = 100 μm.

We assessed the levels of UUO-induced renal fibrosis in the α2AP^+/+^ and α2AP^−/−^ mice using Masson trichrome and Sirius red staining. The degree of Sirius red-positive areas in the obstructed kidneys in the α2AP^−/−^ mice was significantly reduced in comparison to that observed in the α2AP^+/+^ mice (Fig. 2A, B).

### The effect of α2AP deficiency on myofibroblast formation and EMT in the obstructed kidneys

**Figure 3:**
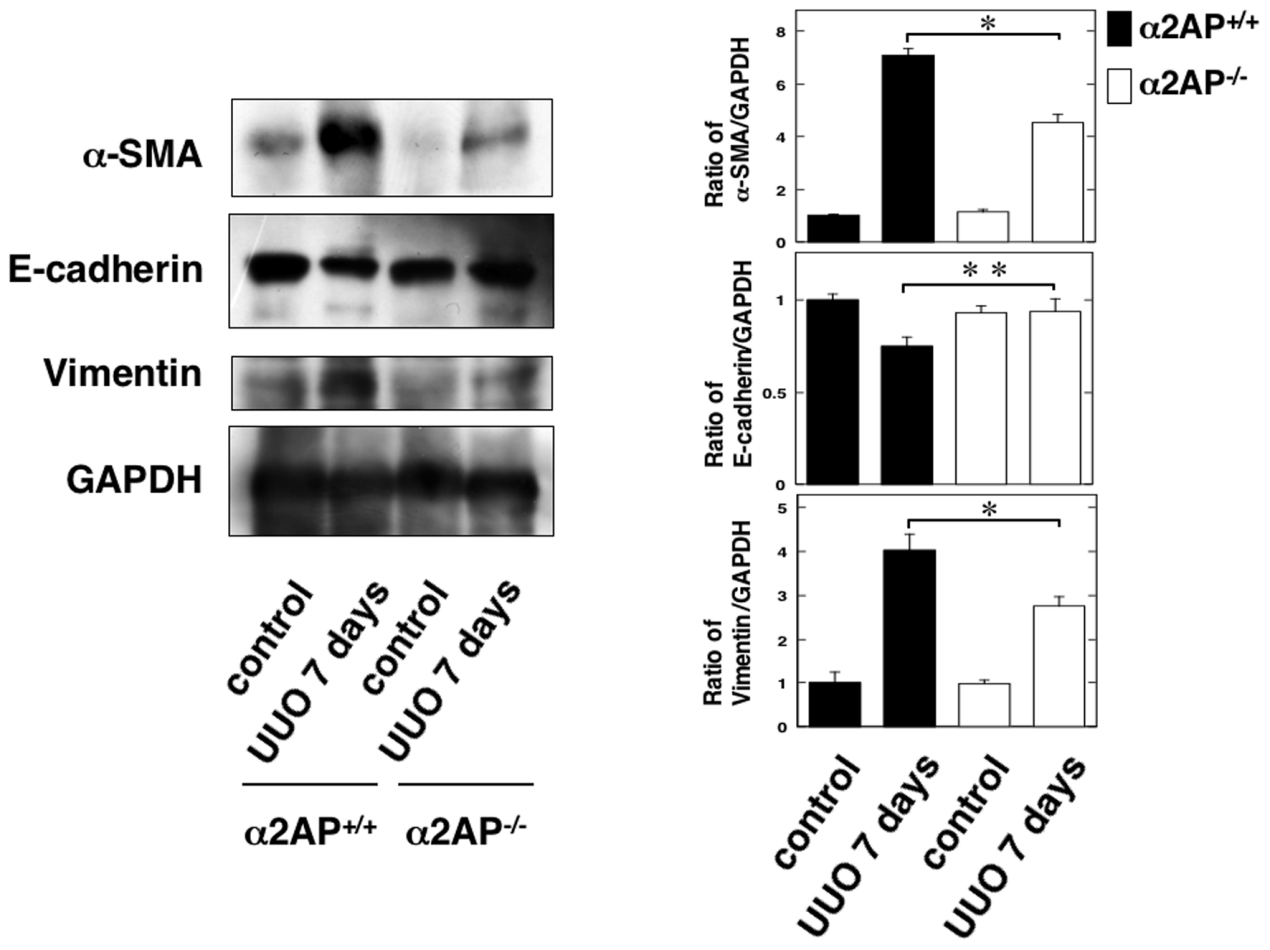
The effect of α2AP deficiency on myofibroblast formation, EMT in the obstructed kidneys. The expression of α-SMA, E-cadherin, vimentin in the kidney of control and 7 days after UUO in the α2AP^+/+^ and α2AP^−/−^ mice was measured by a Western blot analysis. The blots were cropped, and the full-length blots are presented in the supplementary information. The histogram on the right panels shows quantitative representations of α-SMA, E-cadherin, vimentin expression obtained from densitometry analysis (n = 3). The data represent the mean ± SEM. *; *P* < 0.01, **; *P* < 0.05.

To clarify the effects of α2AP deficiency on myofibroblast formation and EMT in obstructed kidneys, we examined the expression of alpha-smooth muscle actin (α-SMA) (a hallmark of the myofibroblast phenotype), two EMT biomarkers (upregulation of vimentin and downregulation of E-cadherin) in the obstructed kidney of the α2AP^+/+^ and α2AP^−/−^ mice (Fig. 3). The degree of α-SMA-positive areas in the obstructed kidneys of the α2AP^−/−^ mice was significantly lower than that observed in the α2AP^+/+^ mice. In addition, the degree of upregulation of vimentin and downregulation of E-cadherin in the obstructed kidneys of the α2AP^−/−^ mice was lower than that observed in the α2AP^+/+^ mice.

### α2AP was associated with myofibroblast formation

**Figure 4:**
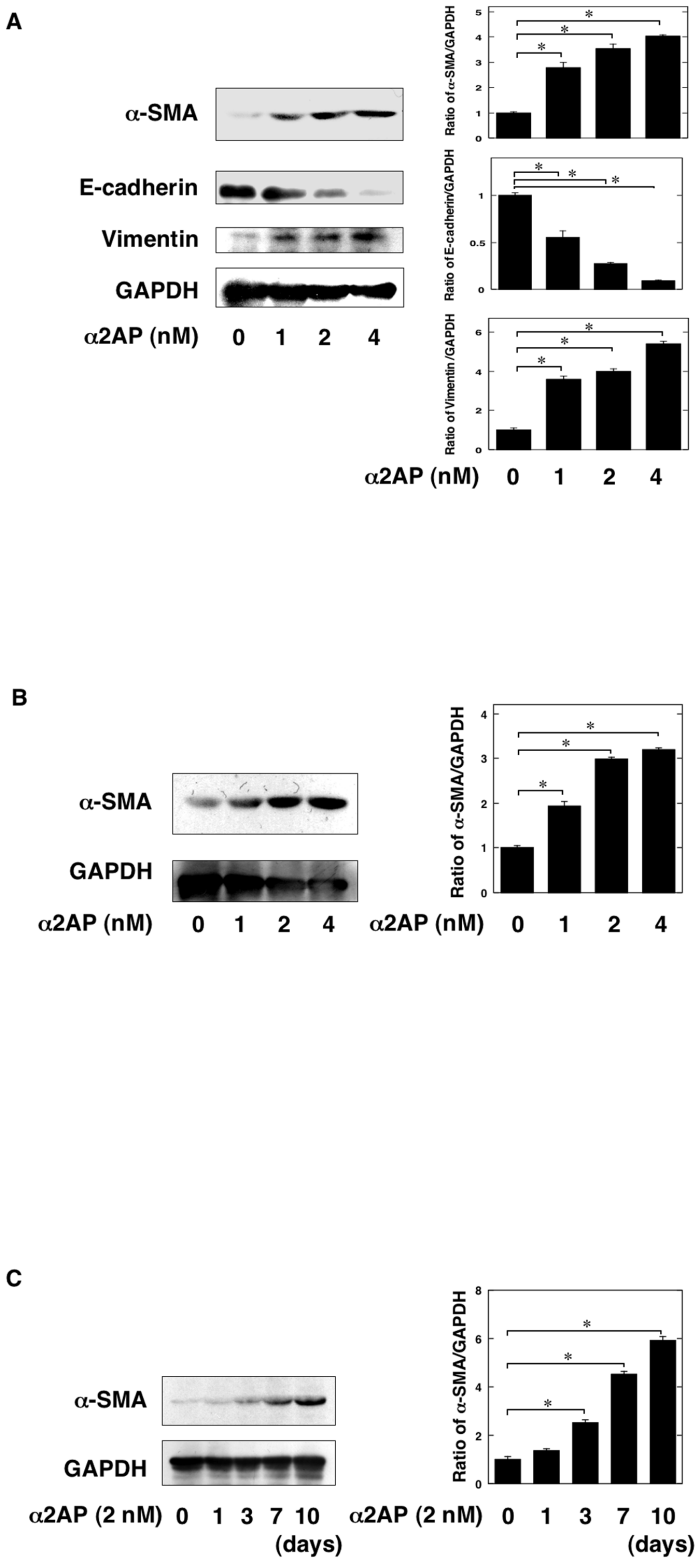
α2AP was associated with myofibroblast formation. (A) The renal tubular epithelial cells were stimulated by α2AP (1, 2, 4 nM) for 24 hours. The expression of α-SMA, E-cadherin, vimentin was measured by a Western blot analysis. The blots were cropped, and the full-length blots are presented in the supplementary information. The histogram on the right panels shows quantitative representations of α-SMA, E-cadherin, vimentin expression obtained from densitometry analysis (n = 3). (B) The renal fibroblasts were stimulated by α2AP (1, 2, 4 nM) for 24 hours. The expression of α-SMA was measured by a Western blot analysis. The blots were cropped, and the full-length blots are presented in the supplementary information. The histogram on the right panels shows quantitative representations of α-SMA expression obtained from densitometry analysis (n = 3). (C) The mesenchymal stem cells were stimulated by α2AP (2 nM) for the indicated periods. The expression of α-SMA was measured by a Western blot analysis. The blots were cropped, and the full-length blots are presented in the supplementary information. The histogram on the right panels shows quantitative representations of α-SMA expression obtained from densitometry analysis (n = 3). The data represent the mean ± SEM. *; *P* < 0.01.

To clarify the role of α2AP on myofibroblast formation, the renal tubular epithelial cells (RTECs), renal fibroblasts and bone marrow-derived mesenchymal stem cells (MSCs) were stimulated by α2AP. α2AP induced the expression of α-SMA, the upregulation of vimentin and the downregulation of E-cadherin in RTECs (Fig. 4A). α2AP also induced the expression of α-SMA in renal fibroblasts (Fig. 4B). Moreover, α2AP induced the expression of α-SMA in bone marrow-derived MSCs (Fig. 4C).

### α2AP induced the production of TGF-β

**Figure 5:**
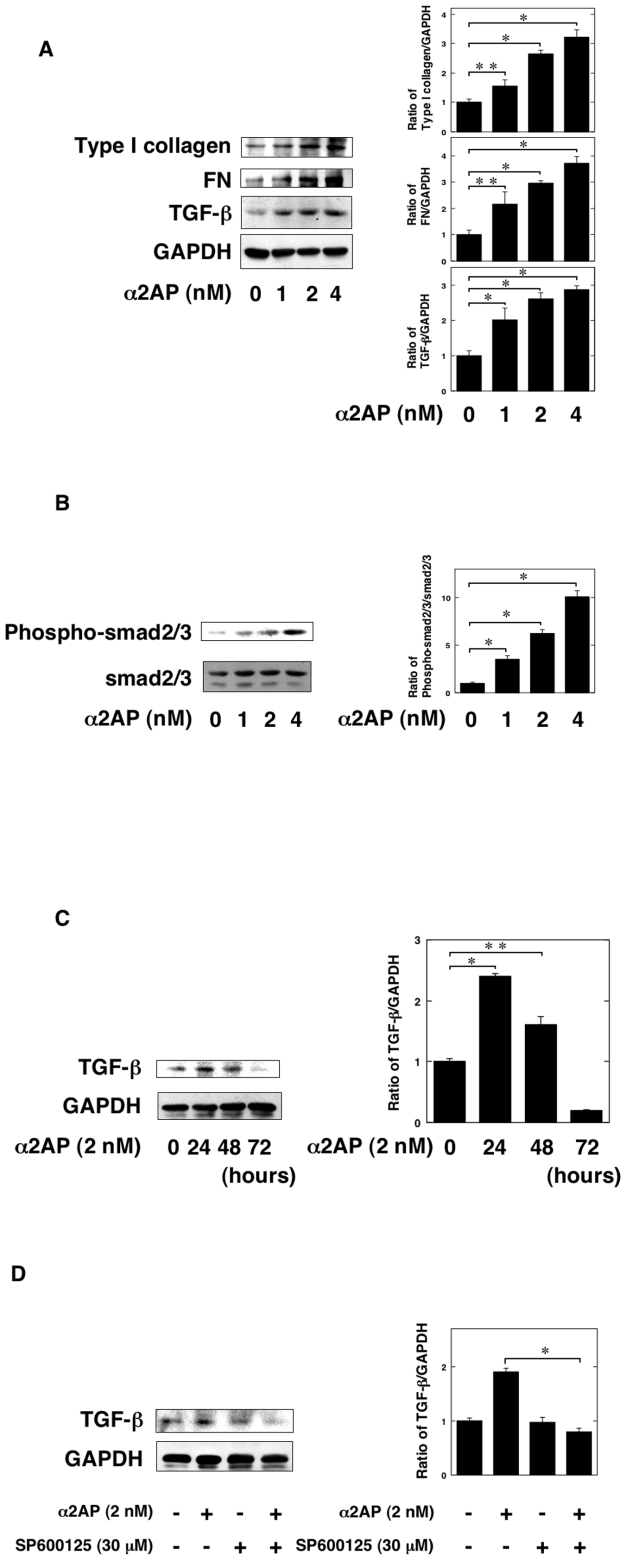
α2AP induced the production of TGF-β. (A) The renal fibroblasts were stimulated by α2AP (1, 2, 4 nM) for 24 hours. The expression of type I collagen, fibronectin (FN), TGF-β was measured by a Western blot analysis. The blots were cropped, and the full-length blots are presented in the supplementary information. The histogram on the right panels shows quantitative representations of type I collagen, FN, and TGF-β expression obtained from densitometry analysis (n = 3). (B) The renal fibroblasts were stimulated by α2AP (1, 2, 4 nM) for 24 hours. Phosphorylation of smad2/3 was measured by a Western blot analysis. The blots were cropped, and the full-length blots are presented in the supplementary information. The histogram on the right panels shows quantitative representations of phospho-smad2/3 expression obtained from densitometry analysis (n = 3). (C) The renal fibroblasts were stimulated with 2 nM α2AP for the indicated periods. The expression of TGF-β was measured by a Western blot analysis. The blots were cropped, and the full-length blots are presented in the supplementary information. The histogram on the right panels shows quantitative representations of TGF-β expression obtained from densitometry analysis (n = 3). (D) The renal fibroblasts were pretreated with DMSO or 30 μM SP600125 for 60 minutes and then were stimulated with 2 nM α2AP for 24 hours. The expression of TGF-β in renal fibroblasts were determined by a Western blot analysis. The blots were cropped, and the full-length blots are presented in the supplementary information. The histogram on the right panels shows quantitative representations of TGF-β expression obtained from densitometry analysis (n = 3). The data represent the mean ± SEM. *; *P* < 0.01. **; *P* < 0.05.

It has known that TGF-β stimulates the formation of myofibroblasts. Therefore, we examined whether α2AP is associated with the production of TGF-β in renal fibroblasts. α2AP induced the production of TGF-β in renal fibroblasts (Fig. 5A). The stimulation of α2AP also induced the production of type I collagen and fibronectin (FN) (Fig. 5A). In addition, the phosphorylation of smad2/3 in α2AP-stimulated cells was increased in a dose-dependent manner (Fig. 5B). The effect of α2AP in the production of TGF-β was seen up to 24 hours after stimulation, after which it decreased (Fig. 5C).

Previously, we demonstrated that α2AP induces the production of TGF-β through JNK pathway^13^. Therefore, we examined whether the JNK pathway is associated with the α2AP-induced TGF-β production by using JNK specific inhibitors (SP600125). SP600125 attenuated the α2AP-induced TGF-β production in renal fibroblasts (Fig. 5D).

## Discussion

The development of renal fibrosis is induced by various factors that stimulate the formation of myofibroblasts, which then synthesize and deposit components of the ECM. However, the mechanisms underlying the formation of myofibroblasts and the development of renal fibrosis are not precisely understood. In this study, we demonstrated the role of α2AP in the formation of myofibroblasts and the development of renal fibrosis.

α2AP has accumulated in the UUO-induced obstructed kidney (Fig. 1). In addition, α2AP deficiency attenuated UUO-induced renal fibrosis (Fig. 2). Plasmin can directly degrade some matrix proteins (fibronectin, laminin, entactin, tenascin, thrombospondin and perlecan), indirectly degrade several other matrix proteins by activating latent metalloproteinases (MMPs) and degrade fibrin, which can serve as a provisional matrix scaffold for the initiation of a fibrotic response^24^. Our previous study demonstrated that α2AP inhibits the plasmin activity^25^, and the inhibition of plasmin may slow ECM degradation and attenuate renal fibrosis. However, it has also been reported that plasmin does not attenuate renal fibrosis^26^. The activation of plasmin is mediated by urokinase-type plasminogen activator (uPA) and tissue-type plasminogen activator (tPA). The inhibition of the system may occur through the neutralization of the plasminogen activators or plasmin, and this neutralization is achieved mainly by the plasminogen activator inhibitor-1 (PAI-1) or α2AP, respectively. uPA also has no antifibrotic activity in renal injury^27^. On the other hand, although PAI-1 or tPA deficiency attenuated the UUO-induced renal fibrosis, both PAI-1 and tPA deficiency had no effect on plasmin activity^28^,^29^. These studies suggest that α2AP deficiency-attenuated renal fibrosis does not result from the enhancement of the plasmin activity due to a deficiency of α2AP and that α2AP itself is associated with the development of renal fibrosis and may function as a local regulator of fibrotic changes.

To clarify the role of α2AP itself in the development of renal fibrosis, we focused the formation of myofibroblasts in obstruct kidney. Myofibroblasts are known to be key effector cells in the development of fibrosis, and myofibroblast formation is promoted by EMT, the differentiation from tissue-resident fibroblasts and bone marrow-derived MSCs^4^,^30^. α2AP deficiency attenuated UUO-induced myofibroblast formation (Fig. 3), and α2AP induced myofibroblast formation in RTECs, renal fibroblasts and bone marrow-derived MSCs (Fig. 4). In addition, α2AP deficiency attenuated UUO-induced the upregulation of vimentin and the downregulation of E-cadherin (Fig. 3), and α2AP induced the upregulation of vimentin and the downregulation of E-cadherin in RTECs (Fig. 4). These data suggest that the myofibroblast formation induced by α2AP is associated with EMT, the differentiation from tissue-resident fibroblatsts and bone marrow-derived MSCs.

It has reported that TGF-β plays a pivotal role in myofibroblast formation, including EMT and the differentiation of fibroblasts. Preiviously, we demonstrated that α2AP induces the production of TGF-β^12^,^13^,^14^. We showed that α2AP induced the production of TGF-β in renal fibroblasts (Fig. 5A). We also showed that α2AP induced ECM protein such as type I collagen and FN. It has known that TGF-β induces myofibroblast formation and the synthesis of ECM protein. TGF-β production induced by α2AP may be associated with myofibroblast formation and ECM deposition. A variety of molecules including plasmin, thrombospondin-1, integrins have been reported as TGF-β activator^31^. Therefore, we examined that the relationship of α2AP and TGF-β activity. The stimulation of α2AP did not interfere with TGF-β signalling (Fig. 5B). Moreover, we showed that the α2AP-induced TGF-β production was associated with JNK pathway (Fig. 5D). JNK pathway is involved in the fibrotic changes such as collagen synthesis and the induction of TGF-β expression^32^,^33^,^34^,^35^, α2AP may mediate the development of renal fibrosis through JNK pathway.

In conclusion, α2AP induces the formation of myofibroblasts via EMT and the differentiation of tissue-resident fibroblasts and bone marrow-MSCs, and mediates the development of renal fibrosis. Our findings may provide new insight into this process, which could eventually lead to the development of new clinical therapies for the prevention of fibrosis.

## Supplementary Material

Supplementary Information Supplementary information
