# Supplementary material for: α2AP mediated myofibroblast formation and the development of renal fibrosis in unilateral ureteral obstruction
Source: Sci Rep. 2014 Aug 6;4:5967. doi: 10.1038/srep05967 (PMC5380014; doi:10.1038/srep05967)

Supplementary information

**α2AP mediated myofibroblast formation and the development of renal fibrosis in unilateral ureteral obstruction**

Running title; The role of α2AP in the development of renal fibrosis

Yosuke Kanno^1^, Eri Kawashita^1^, Akiko Kokado^1^, Hiromi Kuretake^1^, Kanako Ikeda^1^, Kiyotaka Okada^2^, Mariko Seishima^3^, Shigeru Ueshima^2, 4^, Osamu Matsuo^2^, Hiroyuki Matsuno^1^

1: Department of Clinical Pathological Biochemistry, Faculty of Pharmaceutical Science, Doshisha Women’s Collage of Liberal Arts, 97-1 Kodo Kyo-tanabe, Kyoto, 610-0395, Japan.

2: Department of Physiology II. Kinki University School of Medicine, Osaka-sayama, Japan.

3: Department of Dermatology, Gifu University Graduate School of Medicine, Yanagido Gifu, Japan

4: Department of Food Science and Nutrition, Kinki University School of Agriculture, Nara, Japan

All correspondence for Yosuke Kanno:

Requests for offprints should be addressed to Ph.D. Yosuke Kanno, Dept. of Clinical Pathological Biochemistry, Faculty of Pharmaceutical Science, Doshisha Women’s Collage of Liberal Arts, 97-1 Kodo, Kyo-tanabe 610-0395 Kyoto, Japan.

Phone: +81(Japan) 0774-65-8629

E-mail: [ykanno@dwc.doshisha.ac.jp](mailto:ykanno@dwc.doshisha.ac.jp)

Supplementary Figure legends

Supplementary Figure. 1. Full length blots of Figure 1C

Supplementary Figure. 2. Full length blots of Figure 3

Supplementary Figure. 3. Full length blots of Figure 4A

Supplementary Figure. 4. Full length blots of Figure 4B

Supplementary Figure. 5. Full length blots of Figure 4C

Supplementary Figure. 6. Full length blots of Figure 5A

Supplementary Figure. 7. Full length blots of Figure 5B

Supplementary Figure. 8. Full length blots of Figure 5C

Supplementary Figure. 9. Full length blots of Figure 5D


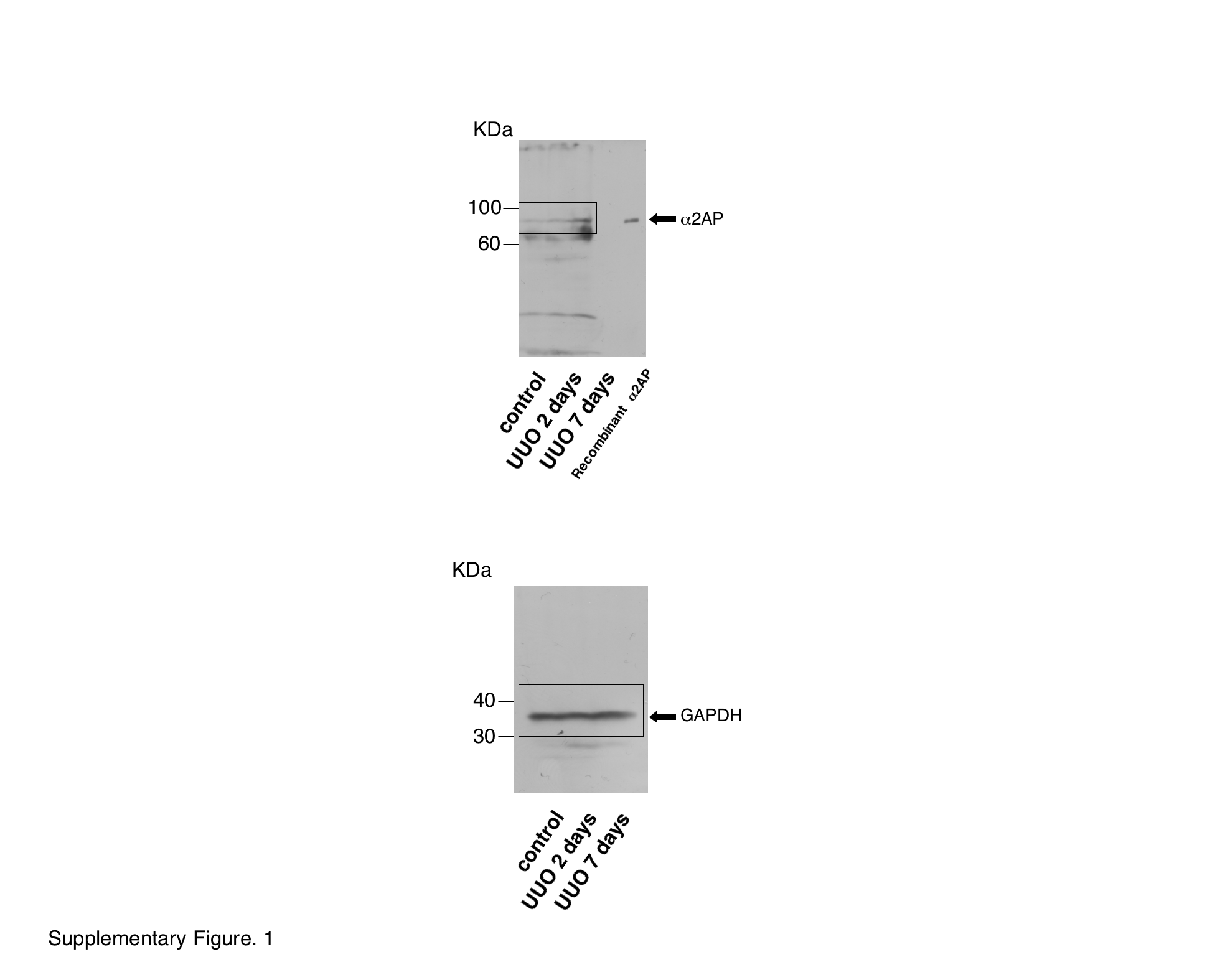


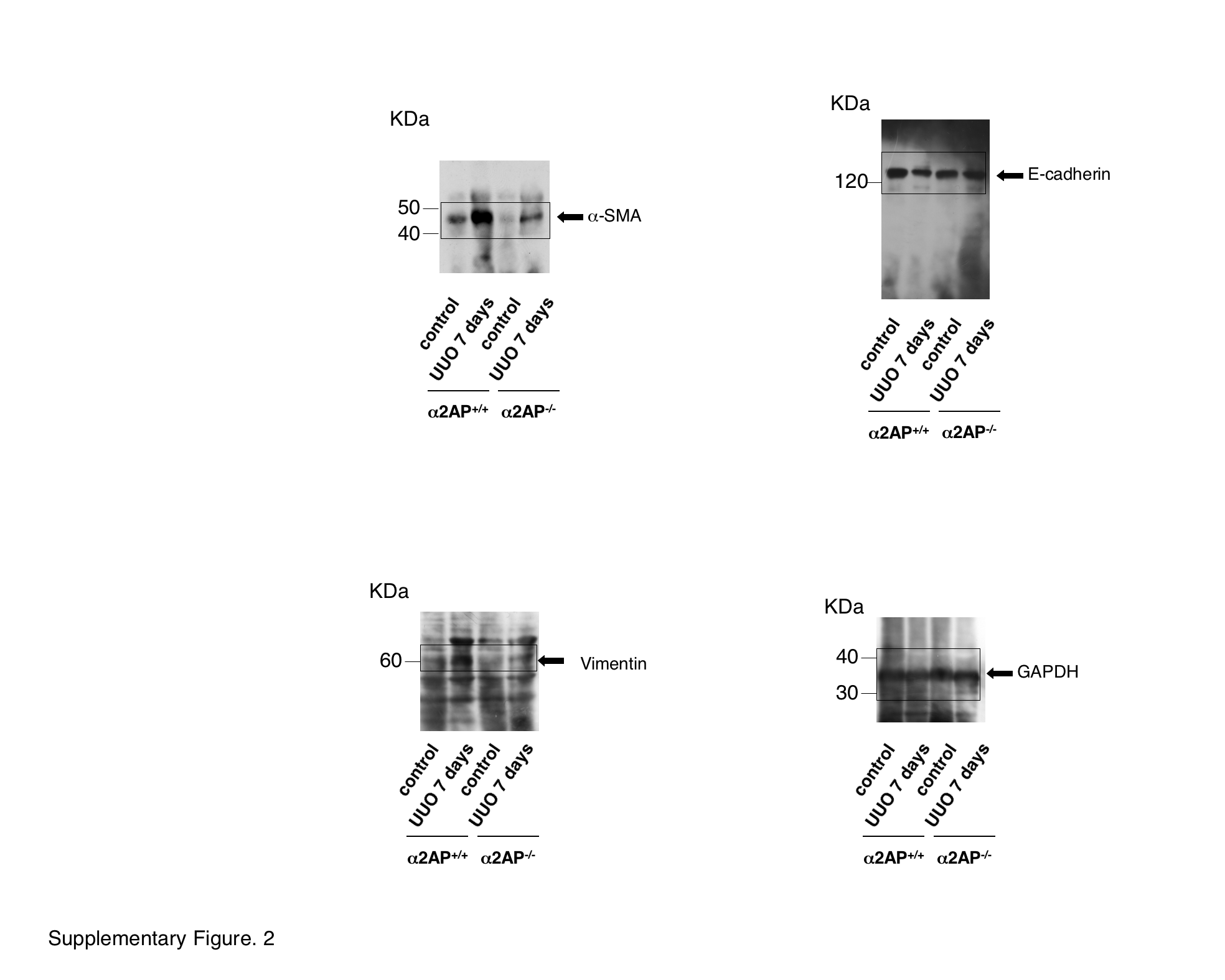


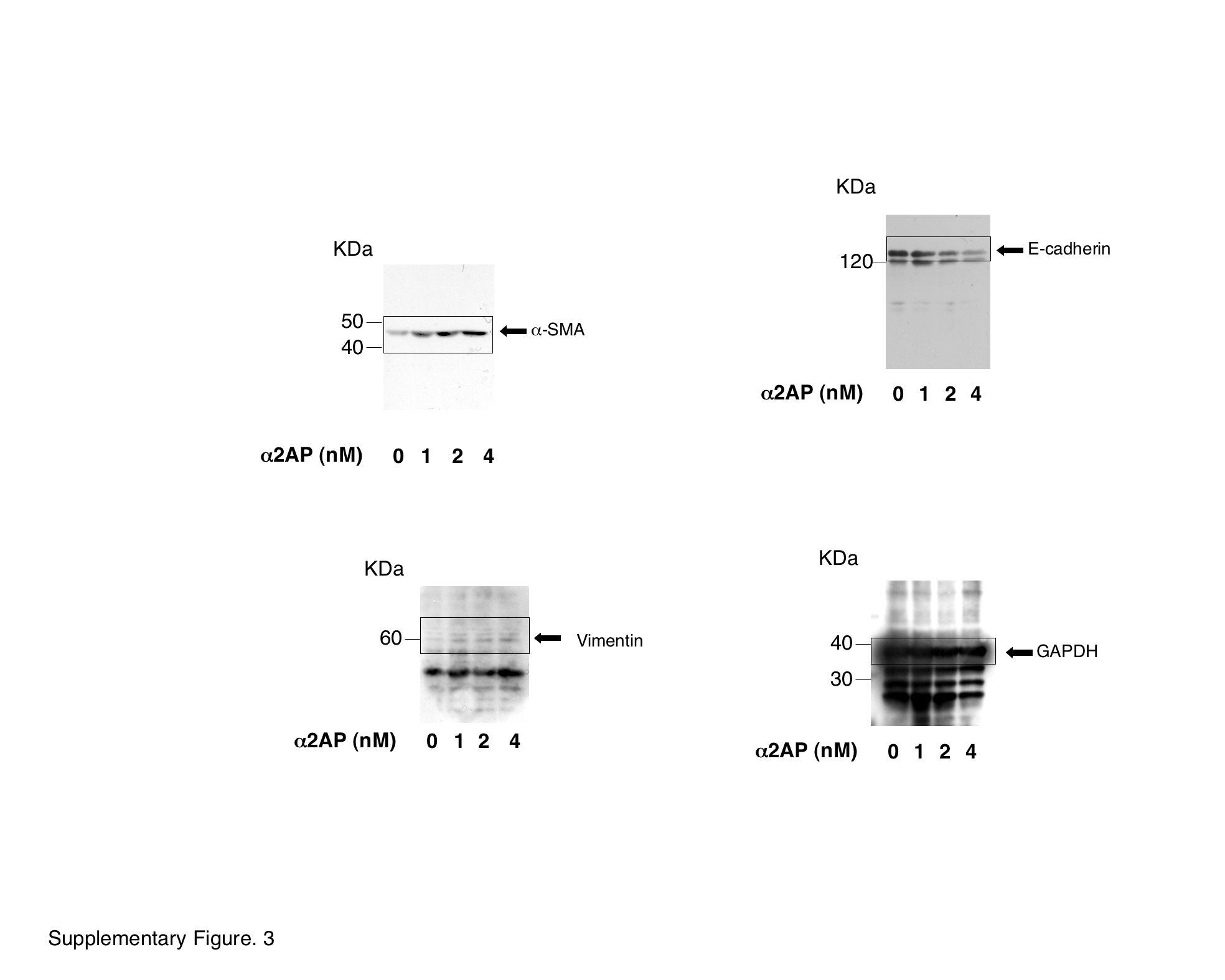


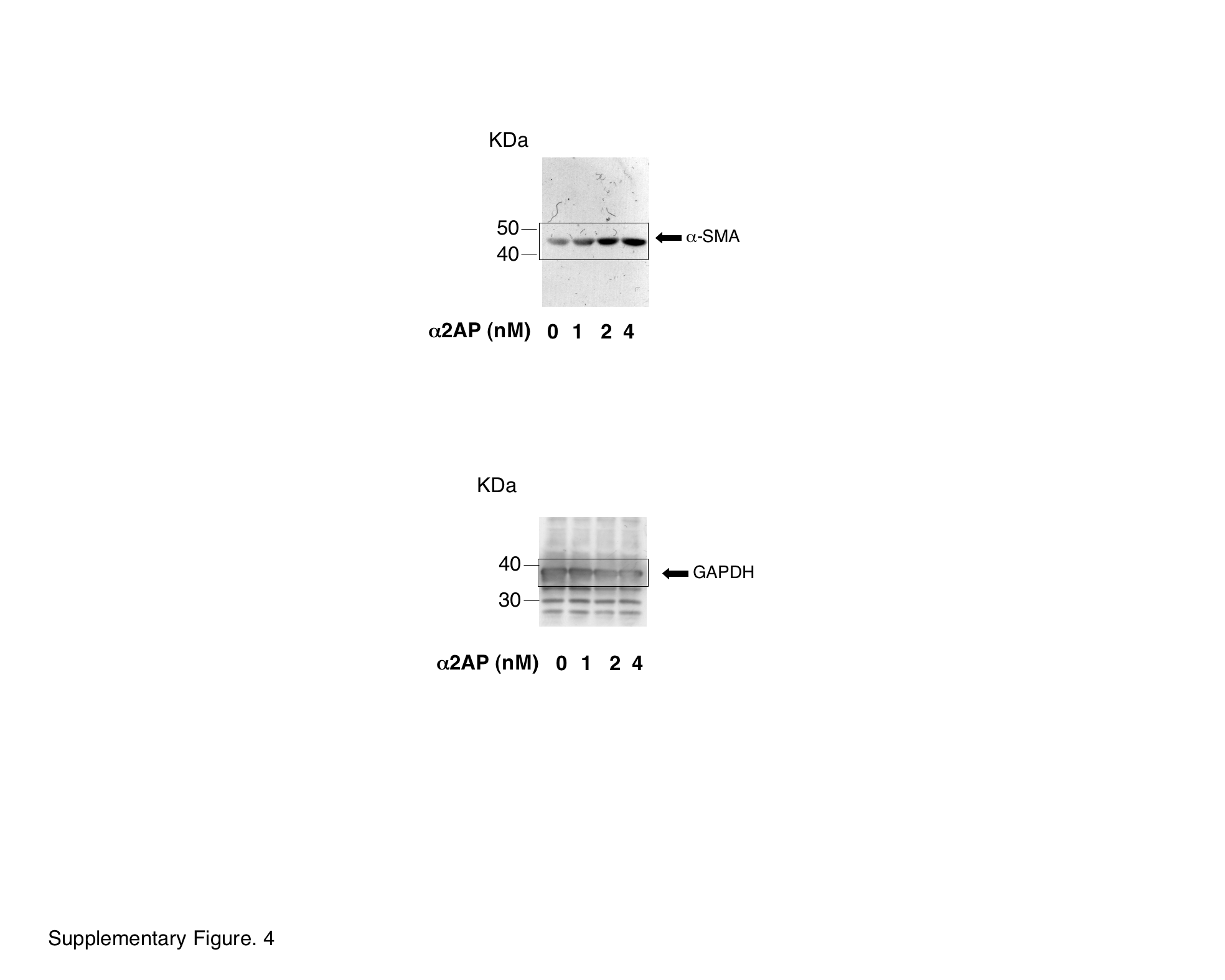


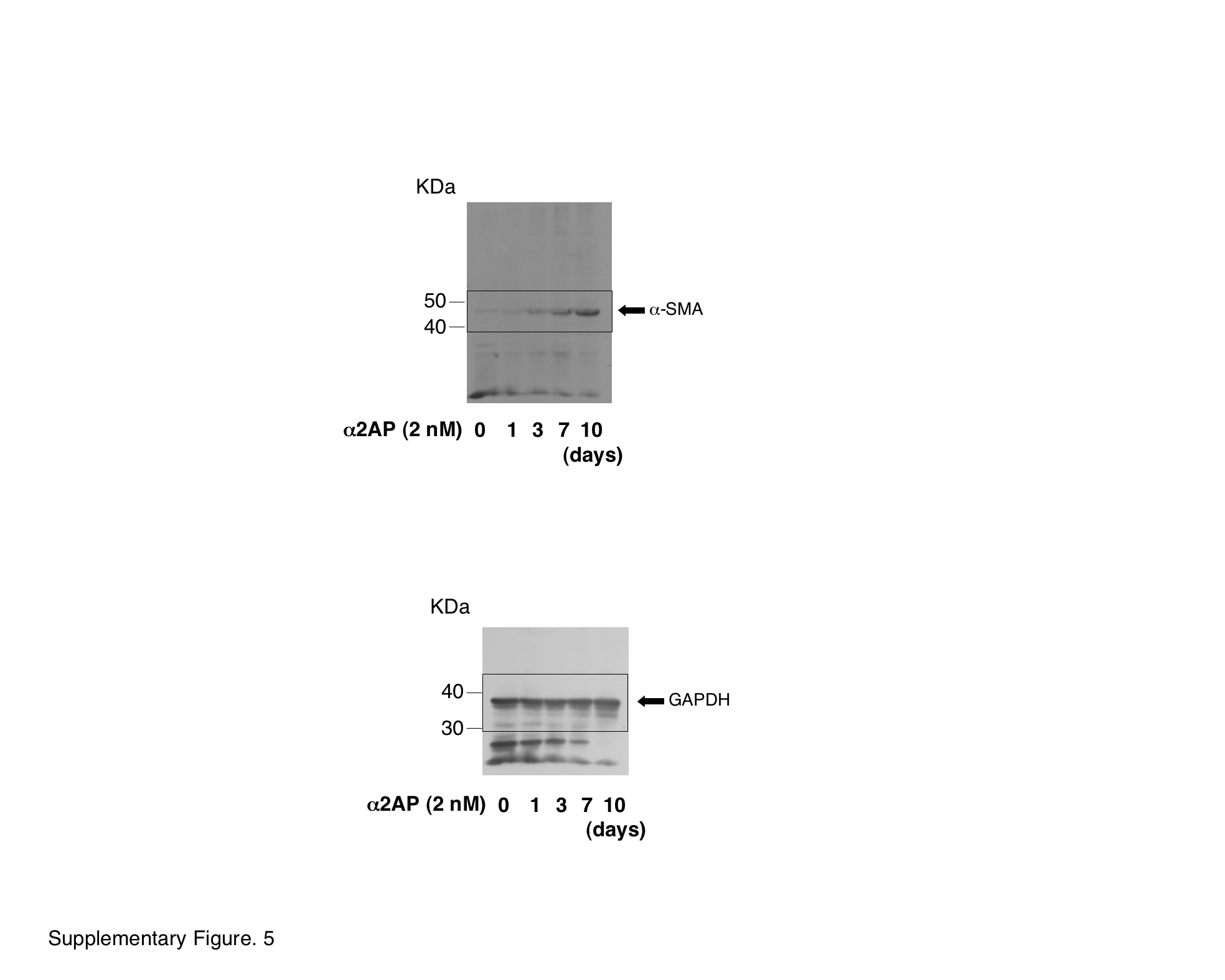


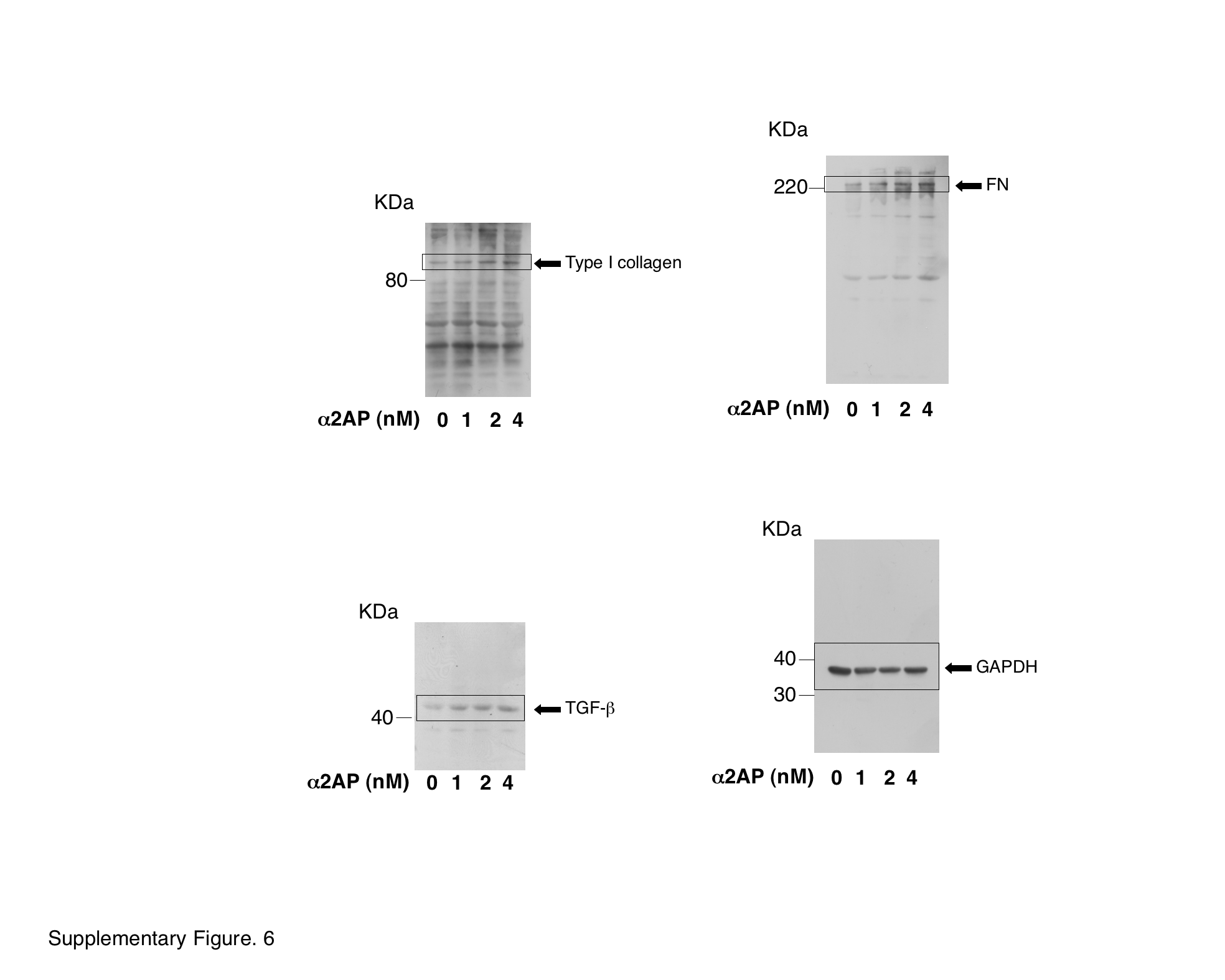


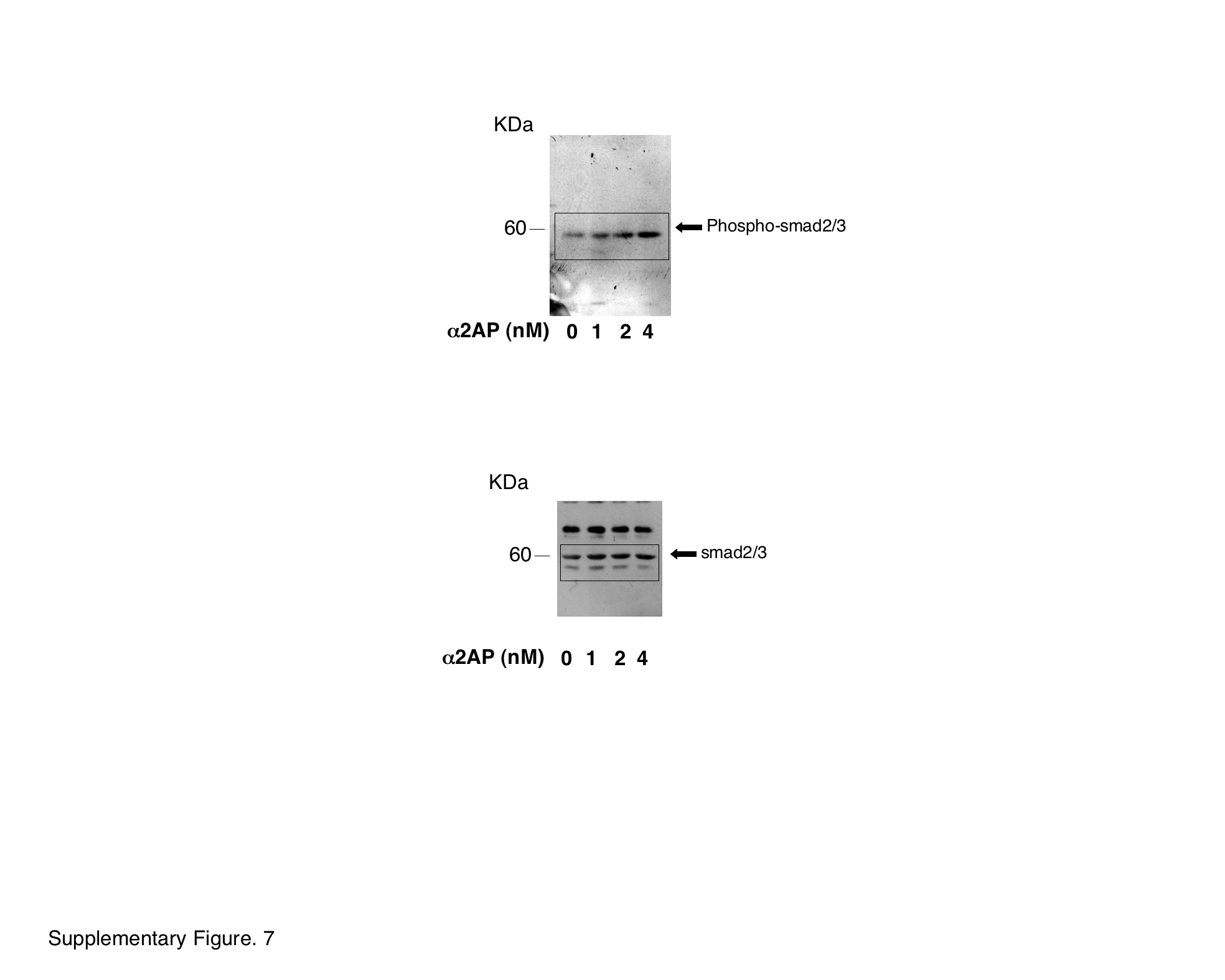


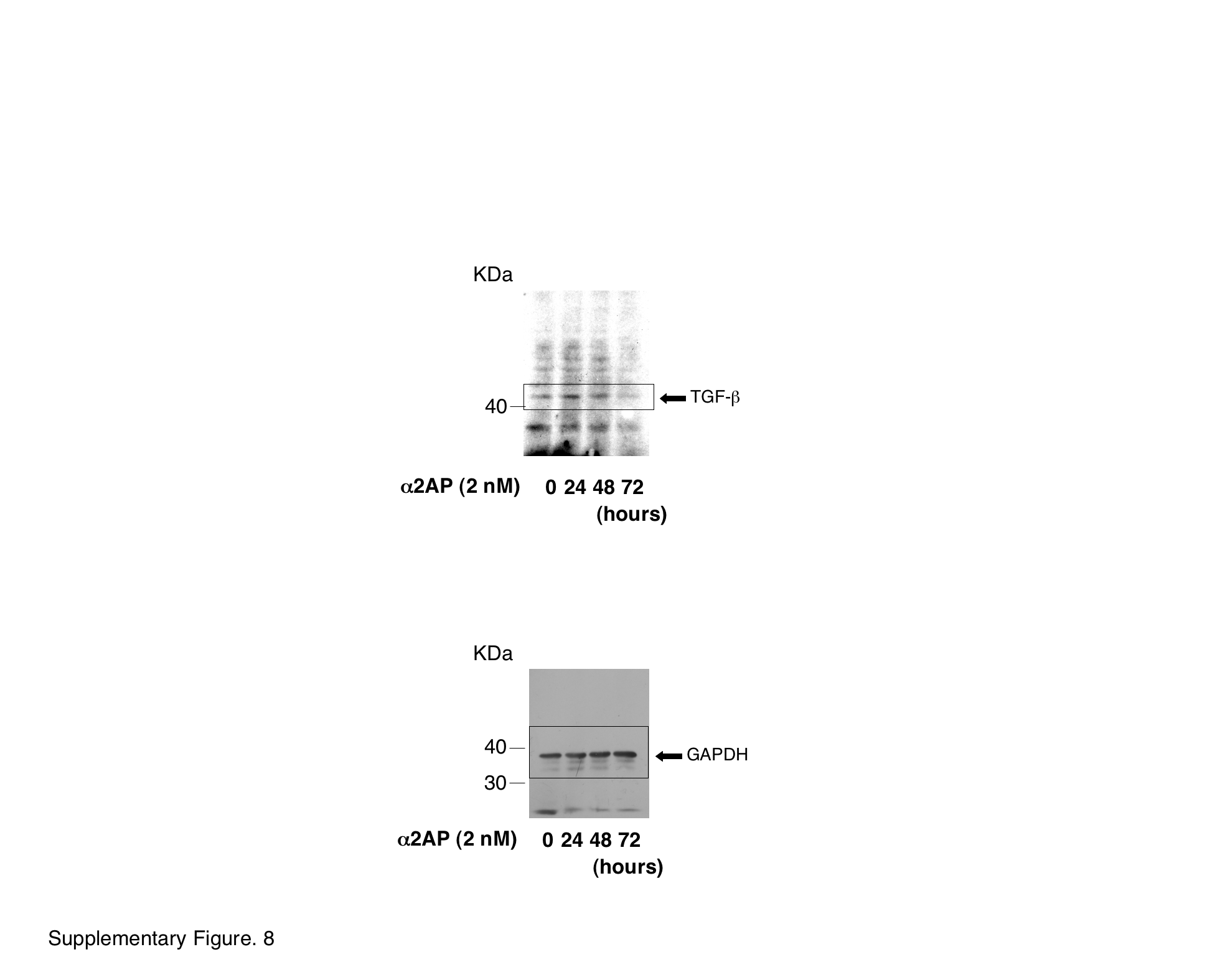


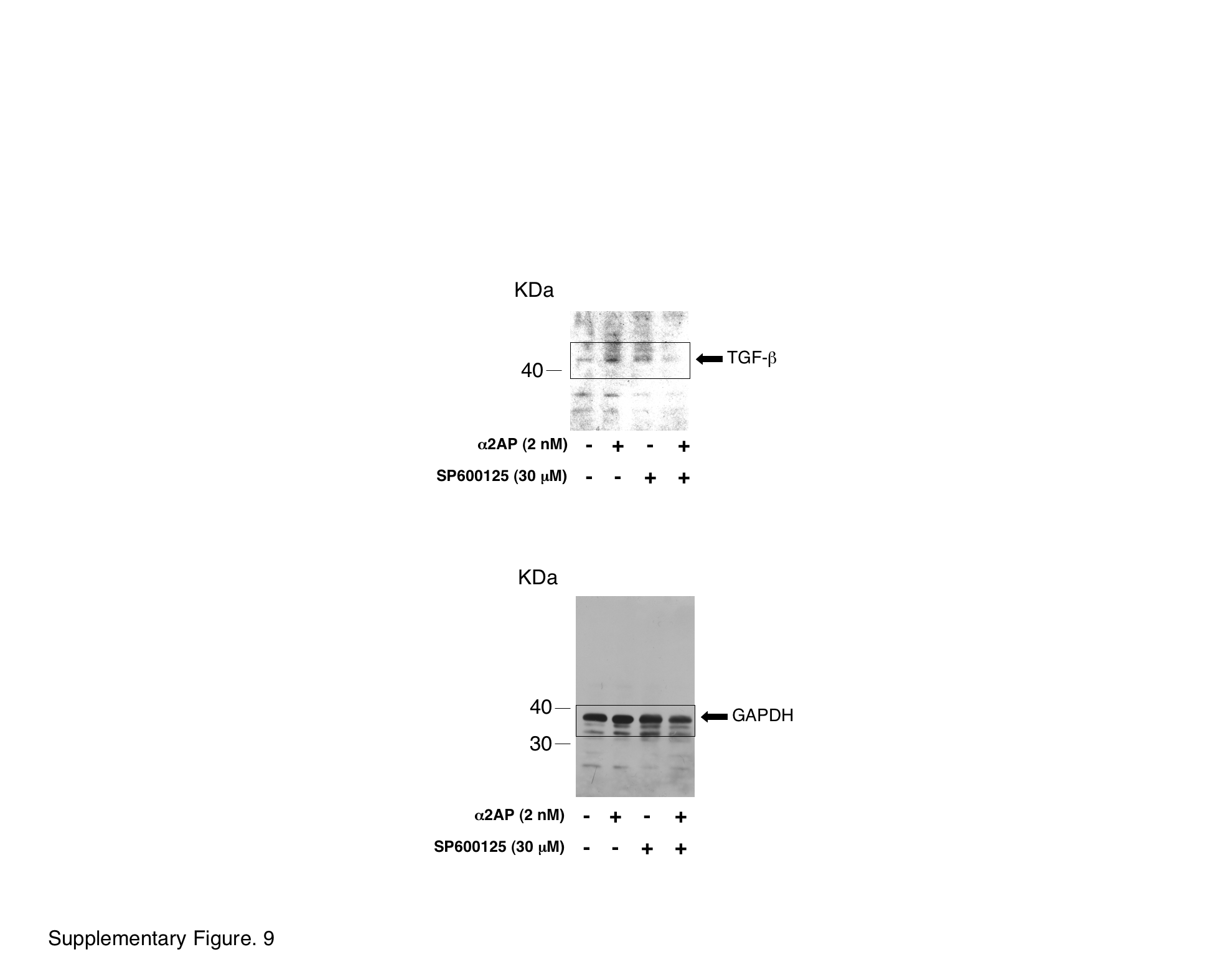

Supplement: Supplementary Information [file srep05967-s1.doc]
